# Validity, consistency and network structure of the World Cancer Research Fund/American Institute for Cancer Research Screener in young adults

**DOI:** 10.3389/fnut.2026.1764935

**Published:** 2026-07-16

**Authors:** Mar Nafría, Alice Chaplin, Lara Prohens, Marga Morey, Antoni Estelrich, Dora Romaguera, Albert Sesé

**Affiliations:** 1 Nutritional Epidemiology and Cardiovascular Fisiopathology (NUTRECOR), Health Research Institute of the Balearic Islands (IdISBa), University Hospital Son Espases (HUSE), Palma, Spain; 2 Consorcio CIBER, M.P. Fisiopatología de la Obesidad y Nutrición (CIBEROBN), Instituto de Salud Carlos III (ISCIII), Madrid, Spain; 3 Department of Psychology, Universitat de les Illes Balears (UIB), Palma, Spain

**Keywords:** cancer prevention, lifestyle assessment, network analysis, public health, validation, young adults

## Abstract

**Introduction:**

The WCRF/AICR Screener is a validated tool for assessing adherence to the World Cancer Research Fund/American Institute for Cancer Research cancer prevention guidelines. This study aimed to evaluate its validity among young adults and examine connections between dietary variables using network analysis.

**Methods:**

Young adults (*n* = 112) were recruited (mean age 24.9 years; 70.5% women) and completed the screener alongside validated assessments: bioelectrical impedance analysis, anthropometry, accelerometry, a 4-week Ecological Momentary Dietary Assessment via smartphone app, and a food frequency questionnaire. For validation, agreement [cross-classification (%), the Kappa coefficient (κ), Spearman correlation (*r*), and linear regression] was determined. Dietary network structure was evaluated using the EBICglasso analysis.

**Results:**

The WCRF/AICR Screener showed moderate-to-strong validity (*r* = 0.57) and good test-retest consistency (*r* = 0.73). Exploratory network analysis revealed proximity between healthier foods and separately less healthy ones. It also suggested that sugar-sweetened drinks was strongly connected to multiple dietary variables, with potential influence on the overall network. In contrast, alcohol intake was relatively isolated from the rest of the variables.

**Discussion:**

These observations support the use of the Screener for assessing cancer prevention recommendations in healthy young adults and generate exploratory hypotheses on dietary interrelationships that require confirmation in larger and more heterogeneous samples.

## 1 Introduction

Cancer is estimated to affect approximately 40% of individuals throughout their lifetime (1), and modifiable lifestyle habits have been linked to the development of 30%−50% of all cancer cases (2, 3). Adherence to the 2018 World Cancer Research Fund/American Institute for Cancer Research (WCRF/AICR) Cancer Prevention Recommendations, which focus on body composition, physical activity, and diet, has been associated with a reduced risk of developing cancer and improved survival (4–6). Adherence to these recommendations can be evaluated using both the WCRF/AICR Score, which was developed for its use in epidemiological studies (7), and the WCRF/AICR Screener (8), which enables a rapid assessment in both research and clinical settings. The WCRF/AICR Screener was validated by our group in an older Spanish population (9) using data based on self-reported collection methods for diet and physical activity, which could introduce a certain degree of bias.

Young adults remain an underrepresented group in cancer prevention strategies, even though young adulthood is a critical period for establishing long-term health behaviors (10). Understanding the validity of the WCRF/AICR Screener in a younger population and examining how its individual components are interconnected could be of interest in public health interventions. In this sense, the use of network analysis remains underexplored in nutritional epidemiology, yet may offer insight into the relationships among dietary variables and could help guide generate hypotheses for prevention strategies (11).

The aim of this study was to further validate the WCRF/AICR Screener in young adults using a novel dietary assessment of diet (12), alongside an objective measure of physical activity (13). Additionally, we aimed to preliminarily explore how the dietary components of the WCRF/AICR Screener are interconnected through network analyses.

## 2 Materials and methods

### 2.1 Study population

Participants (*n* = 112) were recruited though convenience sampling from the University of the Balearic Islands (Palma, Spain), and included students and staff. The sample size was considered suitable for instrument validation and acceptable for exploratory network analyses involving a limited number of nodes (14). Inclusion criteria was: ≥18 and ≤ 45 years of age; absence of language barriers; ability to use digital tools and access to a smartphone with Internet access; and abilityable to attend all visits. Exclusion criteria included pregnancy, and/or an illness that severely affected their diet.

All participants provided written informed consent before the start of the study. The study was approved by the Ethics Committee of the University of the Balearic Islands (325CER23).

### 2.2 Study design and data collection

A cross-sectional study design was used to collect data (Supplementary Figure S1). During the study visit, participants completed the WCRF/AICR Screener together with other questionnaires to collect sociodemographic, dietary, and quality of life data. Body composition was also assessed (further described below). Participants were provided with an accelerometer for 7–10 days and received instructions on how to use the Ecological Momentary Dietary Assessment (EMDA) mobile application over the following 4 weeks. Participants completed the WCRF/AICR Screener again after 10 days for short-term consistency (test-retest analysis). All assessments are described in detail below.

### 2.3 WCRF/AICR Screener

Screener development and main features have been described elsewhere (8) (Supplementary Table S1/English version and Supplementary Table S2/Spanish version). Briefly, it evaluates seven of the ten 2018 WCRF/AICR Cancer Prevention Recommendations and is structured around three dimensions: (1) Body composition; (2) Physical activity; and (3) Diet and alcohol. Anthropometric and physical activity data are collected through open-ended questions, whereas dietary intake is assessed *via* close-ended questions. Each recommendation is scored using the 2018 WCRF/AICR Score criteria: 1 point for fully meeting the recommendation; 0.5 for partially meeting it, and 0 for not meeting it (7). The total score ranges from 0 to 7. Participants completed the WCRF/AICR Screener without assistance and were asked to self-measure their WC using a tape provided by the research team and follow the instructions included in the WCRF/AICR Screener. Self-measured WC was used only for the WCRF/AICR Screener score.

### 2.4 Validated body composition measurement

Weight, height and WC were determined twice by trained health professionals to construct the validated scores. Weight was measured using bioimpedance (BIA) (*Tanita BC-148*, Tanita, Netherlands), recorded to the nearest 100 g, and height was measured using a stadiometer (ADE MZ10017, ADE, Germany) to the nearest 1 mm. WC was measured with a measuring tape to the nearest 1 mm, according to the International Society for the Advancement of Kinanthropometry (ISAK) procedures (15). Body mass index (BMI) was calculated as weight (kg)/height (m)^2^.

### 2.5 Validated physical activity measurement

Physical activity was evaluated using an accelerometer (GENEActiv, Activinsights Ltd, United Kingdom), according to previously published protocols (13, 16). Briefly, participants were asked to wear the accelerometer on their non-dominant wrist for 7–10 days. The GENEActiv device was set to capture and store accelerations at a sampling frequency of 40 Hz (17). Accelerometry data were analyzed using RStudio [version 4.3.2 (2023-10-31)] and the GGIR package (version 3.0–9). Data extracted from the GENEActiv device were processed in bouts of at least 1 min, and categorized according to Hildebrand (18). These cut-offs were expressed in milligravity units (mg) as follows: inactive time during waking hours was defined as < 40 mg, equivalent to < 1.5 metabolic equivalents (METs); light physical activity (LPA) as ≥40 mg and < 100 mg, equivalent to 1.5–3 METs; and moderate-to-vigorous physical activity (MVPA) as ≥100 mg, equivalent to >3 METs. Participants with fewer than 4 valid wear days recorded after data processing were excluded due to insufficient data reliability.

### 2.6 Validated dietary assessment

To assess dietary intake, an EMDA was used together with an adapted food frequency questionnaire (FFQ).

#### 2.6.1 Ecological momentary dietary assessment

Habitual intake was measured by 24 repeated random 2-h recalls (hR) (prompts) over a 4-week period using Traqq^®^, an EMDA app (iOS/Android) (19) that uses a 2hR methodology previously validated for the assessment of energy, nutrient, and food group intake. First, a scheme of consecutive 2hRs covering a full day was created (on average eight 2hRs). The night period was assessed using one late evening (10 pm) and early morning (6 am) 2hR notification. Next, each time slot in this full-day scheme was randomly distributed three times over the 4-week period, twice over weekdays and one during a weekend day, resulting in a total of 24 prompts. This was intended to ensure optimal coverage of the habitual intake and was based on the traditional use of three 24 h recalls. At the end of data collection, the foods and beverages reported were converted into dietary intake estimates, and food items relevant to cancer prevention were selected and converted into portions for comparison with the WCRF/AICR Screener.

To ensure data quality, two exclusion criteria were applied: (1) Participants were required to respond to at least 21 prompts; and (2) Dietary data were screened following the Willett inclusion/exclusion criteria (20) to identify under- or over- reporters of energy intake.underreporting < 500 kcal/day/women or < 800 kcal/day/men, and overreporting >3,500 kcal/day/women or > 4,000 kcal/day/men (20).

#### 2.6.2 Food frequency questionnaire

An adapted, validated, semi-quantitative FFQ for the Spanish population (21–23) was used to measure consumption of food items relevant to cancer prevention, based on the WCRF/AICR recommendations. This adaptation consisted in retaining and adding relevant food items, resulting in a total of 91 items (Supplementary Table S3).

### 2.7 Covariates

Sociodemographic characteristics, including sex, gender, nationality, age, culinary skills, living arrangements, educational level, illness, smoking habits, food intolerances and special dietary requirements, were obtained using in-house questionnaires. Quality of life was assessed using the 26-item WHOQOL-BREF (24). Body composition measures (total fat percentage, visceral fat, and lean mass) were obtained using BIA (*Tanita BC-148*, Tanita, Netherlands).

## 3 Statistical analyses

### 3.1 Validation

The WCRF/AICR Screener score was compared with two alternative composite scores based on more detailed and objective assessments. *Validated Score-EMDA* was constructed using measured anthropometry, accelerometer-based physical activity data, and dietary intake assessed through the EMDA mobile application. *Validated Score-FFQ* used the same anthropometric and physical activity measurements, but dietary intake was assessed using the FFQ.

The validation study population was divided into tertiles based on their WCRF/AICR Screener score: low adherence (tertile 1, T1), medium adherence (T2) and high adherence (T3). Baseline characteristics are shown as numbers and percentages, means and standard deviations, or medians and interquartile ranges (IQR). Normality of data was assessed using the Anderson-Darling test. Differences across groups were assessed using the chi-squared tests (X^2^), a one-way ANOVA or a Kruskal-Wallis test, as appropriate.

Agreement between the overall WCRF/AICR Screener score and the reference scores was assessed as follows (25): cross-classification (percentage of participants scoring the same for both scores); Cohen's weighted kappa statistic [κ values >0.8 indicate almost perfect agreement; 0.61–0.80 substantial agreement; 0.41–0.60 moderate agreement; 0.21–0.40 fair agreement; and ≤ 0.20 slight agreement (26)]; the Spearman correlation coefficient (*r*) for general agreement; and the coefficient of determination (*R*^2^) through linear regression, using the Screener score as the predictor variable and the validated score as the response variable. A separate linear regression was performed to assess proportional bias, where the difference between the two scores was used as the dependent variable and their mean as the independent variable. Analyses were carried out using Stata, version 17.0 (StataCorp LLC), and the threshold of significance was set at 0.05.

### 3.2 Network analysis

Network analysis is a method that can be used to examine interconnections between lifestyle variables (27), whereby nodes represent individual lifestyle variables, and edges represent statistical connections between them (detailed description of the *a priori* network can be found in Supplementary Table S4).

The network structure was estimated using the EBICglasso procedure (28). Network stability and accuracy were assessed using 1,000 bootstrap analyses to estimate the 95% confidence interval weights around the partial connections between variables. Centrality analysis was conducted to identify the most influential variables in the network. We used the centrality plot to estimate three centrality indices: strength, closeness, and betweenness (29). A case-dropping bootstrap based on 1,000 non-parametric bootstrap samples, evaluated the stability of centrality indices. To assess the network's global structure, we computed two small-worldness indices: S.rand and S.HG: The S.rand index compares the network to a random network with similar characteristics, while the S.HG index compares it to a hierarchical graph, which reflects more realistic structures. A value of S>1 suggests that the network has a high level of clustering and short connections between variables, which are typical characteristics of small-world networks. In contrast, S < 1 represents a random network. Network estimation was performed using the bootnet, NetworkToolbox (28), and qgraph (30) packages in R program (31).

## 4 Results

### 4.1 Study population characteristics

**Figure 1:**
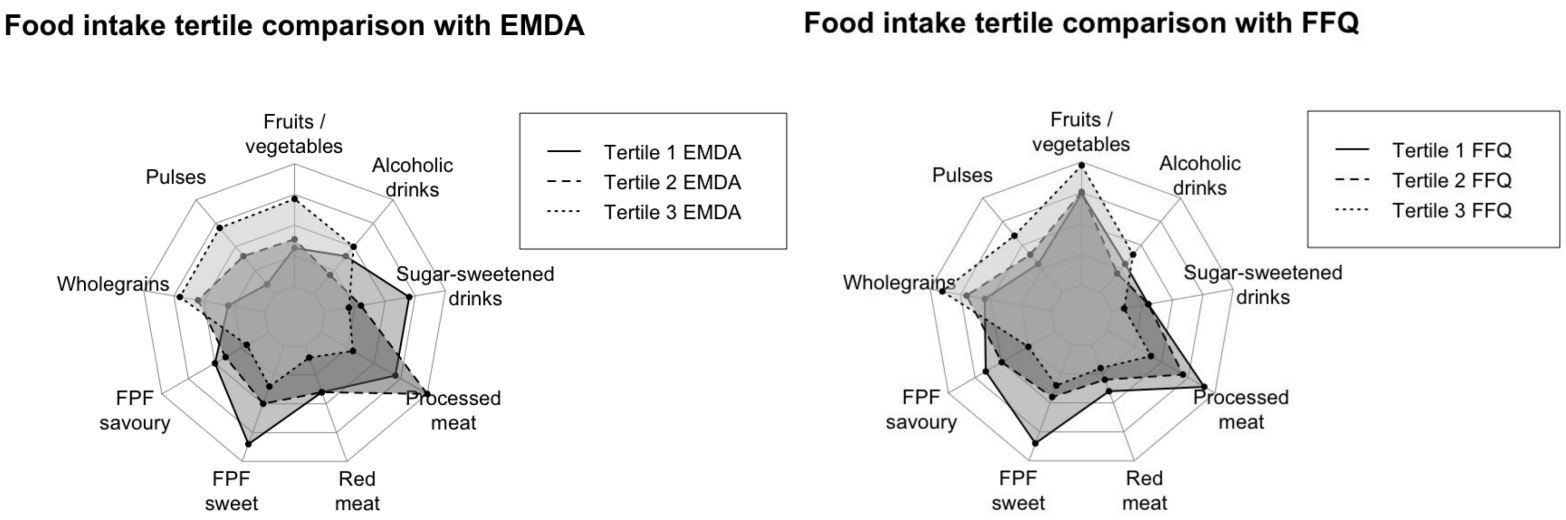
Dietary intake measure through EMDA and FFQ of food groups included in the WCRF/AICR Screener. Food intake is presented in tertiles. Ranges are presented as follows [max-min]: Fruits/vegetables: [7-0]; Pulses: [1-0]; Wholegrains: [2-0]; FPF savoury: [2-0]; FPF sweet: [2-0]; Red meat: [1-0]; Processed meat: [1-0]; Sugar-sweetened drinks: [1-0]; Alcoholic drinks: [1-0]. EMDA, Ecological Momentary Dietary Assessment; FFQ, Food Frequency Questionnaire; FPF savoury, Fast and processed food – savoury; FPF sweet, Fast and processed food – sweet; Fruits/vegetables, Fruits and vegetables.

A total of 112 participants were recruited (see Supplementary Figure S2 for a detailed flow chart). All participants completed study questionnaires, and anthropometry measurements were obtained for all. Incomplete accelerometry data (*n* = 11) was excluded from analysis. A total of 109 participants completed the 4-week EMDA period and 30 were excluded from the analysis due to insufficient responses (*n* = 23), underreporting (*n* = 1), or overreporting (*n* = 6). Distribution of participant characteristics according to the WCRF/AICR Screener score is shown in Table 1. Participants with higher scores had lower BMI and WC, lower body fat mass, lower visceral fat, and higher physical activity levels. Furthermore, they were more likely to cook for themselves, studied a university degree related to health sciences, had a better self-perceived quality of life, did not smoke, and a lower proportion lived with their parents. Dietary consumption is shown in Figure 1 (data in Supplementary Table S5).

**Table 1:** Characteristics of the study population according to tertiles of the WCRF/AICR screener.

Descriptive characteristics	All participants	T1 low adherence	T2 medium adherence	T3 high adherence	*P* value^a^
[Range]		[1, 3.8]	[4, 4.75]	[4.83, 7]	
*n*	112	38	37	37	
Scores					
WCRF/AICR Screener score [0–7]	4.4 (1.2)	3.1 (0.6)	4.3 (0.2)	5.7 (0.6)	<0.001
Validated score-EMDA [0–7]	4.75 (0.9)	4.2 (0.8)	4.7 (0.7)	5.4 (0.6)	<0.001
Validated score*-*FFQ [0–7]	3.6 (0.9)	3.0 (0.6)	3.4 (0.7)	4.4 (0.9)	<0.001
Sociodemographic data					
Women, *n* (%)	79 (70.5)	29 (76.3)	23 (62.2)	27 (73.0)	0.374
Age (years)	24.9 (5.8)	24.2 (6.3)	24.9 (5.7)	25.6 (5.3)	0.302
Pursuing a degree, *n* (%)	64 (57.1)	26 (68.4)	22 (59.5)	16 (43.2)	0.083
Studying health sciences, *n* (%)	64 (57.1)	16 (42.1)	23 (62.1)	25 (67.6)	0.063
Living with their parents, *n* (%)	59 (52.7)	21 (55.3)	22 (59.5)	16 (43.2)	0.349
Cook for themselves, *n* (%)	67 (59.8)	16 (42.1)	23 (62.1)	28 (75.7)	0.012
Current smokers, *n* (%)	3 (5.3)	2 (10.5)	1 (6.3)	0 (0.0)	0.367
Quality of life [0–100]	60.4 (8.1)	58.4 (8.4)	60.2 (8.0)	62.6 (7.6)	0.056
Body composition					
BMI (kg/m^2^)	23.1 (3.7)	23.7 (4.7)	23.8 (3.4)	21.9 (2.0)	0.039
Waist circumference (cm)	72.9 (9.3)	73.8 (10.2)	74.6 (10.2)	70.3 (6.7)	0.158
Body fat mass (%)	23.6 (9.3)	26.6 (11.1)	23.7 (9.2)	20.4 (6.1)	0.017
Visceral fat (%)	2.5 (2.1)	2.9 (2.4)	2.9 (2.5)	1.8 (1.1)	0.043
Physical activity					
Moderate PA (min/week)	118.8 (43.1)	110.3 (34.7)	122.7 (56.5)	123.0 (34.9)	0.237
Vigorous PA (min/week)	7.6 (7.2)	6.5 (5.1)	6.7 (6.5)	9.5 (9.0)	0.275
Dietary patterns					
Does not follow a specific dietary pattern, *n* (%)	75 (67)	31 (81.6)	21 (56.8)	23 (62.2)	0.129
Vegetarian or vegan, *n* (%)	10 (8.9)	2 (5.3)	3 (8.1)	5 (13.5)	
Other dietary patterns, *n* (%)	27 (24.1)	5 (13.1)	13 (35.1)	9 (24.3)	

Values are presented as means (SD, standard deviation) for continuous variables and n (%) for categorical variables. AC, accelerometry; BMI, body mass index; EMDA, ecological momentary dietary assessment; FFQ, food frequency questionnaire; PA, physical activity; SD, standard deviation; T, tertile; WCRF/AICR, World Cancer Research Fund/American Institute for Cancer Research.

^a^A X2 test, one-way ANOVA, Kruskal-Wallis test or Fisher test was carried out as appropriate. Threshold of significance: *p* < 0.05.

### 4.2 Validation results

Scores obtained for each WCRF/AICR recommendation using the WCRF/AICR Screener showed a strong correlation with those obtained from the *Validated Score-EMDA* (*n* = 71; Table 2). The highest agreement was observed for “Body composition” (93.0%) and the lowest for “Pulses” (43.0%). These findings were consistent with the *Validated Score-FFQ* (*n* = 101), available in Supplementary Table S6.

**Table 2:** Distribution and agreement between scores of each WCRF/AICR recommendation assessed using the screener and the *Validated Score-EMDA* (*n* = 71).

Screener domains	WCRF/AICR Screener Score			Validated score-EMDA			Cross-classification^a^	CWK
Score	0	0.5	1	0	0.5	1		
	*n* (%)	*n* (%)	*n* (%)	*n* (%)	*n* (%)	*n* (%)	(%)	(κ)
Body composition	4 (5.6)	16 (22.6)	51 (71.8)	2 (2.0)	10 (14.1)	59 (83.1)	93.0	0.677
Physical activity	13 (18.3)	8 (11.3)	50 (70.4)	6 (8.5)	44 (62.0)	21 (29.5)	64.8	0.141
Plant-based foods^a^	15 (21.1)	46 (64.8)	10 (14.1)	8 (11.3)	36 (50.7)	27 (38.0)	83.1	0.510
Fruit and vegetables	19 (26.8)	39 (54.9)	13 (18.3)	27 (38.0)	30 (42.3)	14 (19.7)	79.6	0.457
Pulses	7 (9.8)	46 (64.8)	18 (25.4)	69 (97.2)	1 (1.4)	1 (1.4)	43.0	−0.001
Wholegrains	39 (54.9)	18 (25.3)	14 (19.7)	71 (100)	0 (0.0)	0 (0.0)	67.6	0.000
Fast and processed foods	23 (32.4)	21 (29.6)	27 (38.0)	10 (14.1)	20 (28.2)	41 (57.75)	66.9	0.258
Red and processed meat	34 (47.9)	16 (22.6)	21 (29.6)	6 (8.5)	13 (18.3)	52 (73.2)	55.6	0.176
Sugar-sweetened drinks	3 (4.2)	31 (43.7)	37 (52.1)	0 (0.0)	31 (43.7)	40 (56.3)	80.3	0.269
Alcoholic drinks	1 (1.4)	43 (60.6)	27 (38.0)	0 (0.0)	29 (40.9)	42 (59.1)	83.1	0.369

Cross-classification (%) was calculated as the percentage of participants scoring the same both through the WCRF/AICR Screener and validated methods. CWK, Cohen's weighted kappa; EMDA, ecological momentary dietary assessment; WCRF/AICR: World Cancer Research Fund/American Institute for Cancer Research.

^a^Scores for plant-based foods (mean of the individual scores assigned to “Fruit and vegetables”, “Pulses” and “Wholegrains”) were rounded to 0.5.

After exclusions, within the Validated Score-EMDA sample, the mean WCRF/AICR Screener score (4.36 ± 1.15) was slightly lower than the mean EMDA score (4.73 ± 0.89). Conversely, for the Validated Score-FFQ sample, the mean Screener score (4.40 ± 1.18) was higher than the mean FFQ score (3.61 ± 0.89; Table 3).

**Table 3:** Correlation, agreement, and consistency of the overall WCRF/AICR screener.

Statistics	*n* = 71		*n* = 101		*n* = 81	
	WCRF/AICR Screener score	Validated score-EMDA	WCRF/AICR Screener score	Validated score-FFQ	WCRF/AICR Screener (Day 0)	WCRF/AICR Screener score (Day 7–10)
Score (0–7), mean (SD)	4.36 (1.15)	4.73 (0.89)	4.40 (1.18)	3.61 (0.89)	4.49 (1.16)	4.58 (1.09)
Intercept, β0 (SE)^a^	2.60 (0.32)		1.44 (0.26)		1.28 (0.30)	
Regression coefficient, β1 (SE) [95% CI]^b^	0.48 (0.07) [0.34, 0.63]		0.49 (0.06) [0.38, 0.61]		0.67 (0.07) [0.60, 0.86]	
Residual SE	0.69		0.68		0.69	
Coefficient of determination, *R*^2^	0.40		0.42		0.73	
Spearman correlation, r	0.57		0.61		0.73	

^a^Linear regression models were fitted with the validated score as the dependent variable and the WCRF/AICR Screener score as the independent variable for validity analyses. Scores obtained at Day 7–10 were established as the dependent variable and Day 0 score as the independent variable for test-retest analysis.

^b^Regression coefficients (β1) are presented with their SE and 95% CI. β0, intercept; β1, regression coefficient; CI, confidence interval; EMDA, Ecological Momentary Assessment; FFQ, Food Frequency Questionnaire; Residual SE, residual standard error; SD, Standard deviation; SE, Standard error; WCRF/AICR, World Cancer Research Fund/American Institute for Cancer Research.

Regression analysis indicated that each one-point increase in the Screener score was associated with a 0.48-point increase in the *Validated Score-EMDA* and a 0.49-point increase with the *Validated Score-FFQ*. Furthermore, the Screener explained 42% and 40% of the variance in the validated scores, respectively. Spearman correlation coefficients (*r* = 0.57–0.61) also indicated moderate to strong associations.

Agreement between the WCRF/AICR Screener score and the *Validated Score-EMDA* and the *Validated Score-FFQ* were determined (Supplementary Figures S3, S4). When compared to the EMDA-derived score, there was a mean difference of 0.36 (± 1.96 SD: −1.41 to 2.14), whereas the difference of means was −0.78 (± 1.96 SD: −2.55 to 0.99) when analyzing the *Validated Score-FFQ*. In this sense, there was evidence of proportional bias in the regression coefficients, as shown by a significant negative association between the mean score and the difference between scores (*Validated Score-EMDA;* β = −0.32, *p* = 0.006; *Validated Score-FFQ*; β = −0.33, *p* < 0.001). This trend was partially observed in the Bland–Altman plot.

Short-term consistency (test-retest) analysis indicated that the Screener scores (*n* = 81) remained stable (4.49 ± 1.16 vs. 4.58 ± 1.09) with a strong association (*R*^2^ = 0.61) and a good correlation (*r* = 0.73), indicating good temporal consistency.

### 4.3 Network estimation

**Figure 2:**
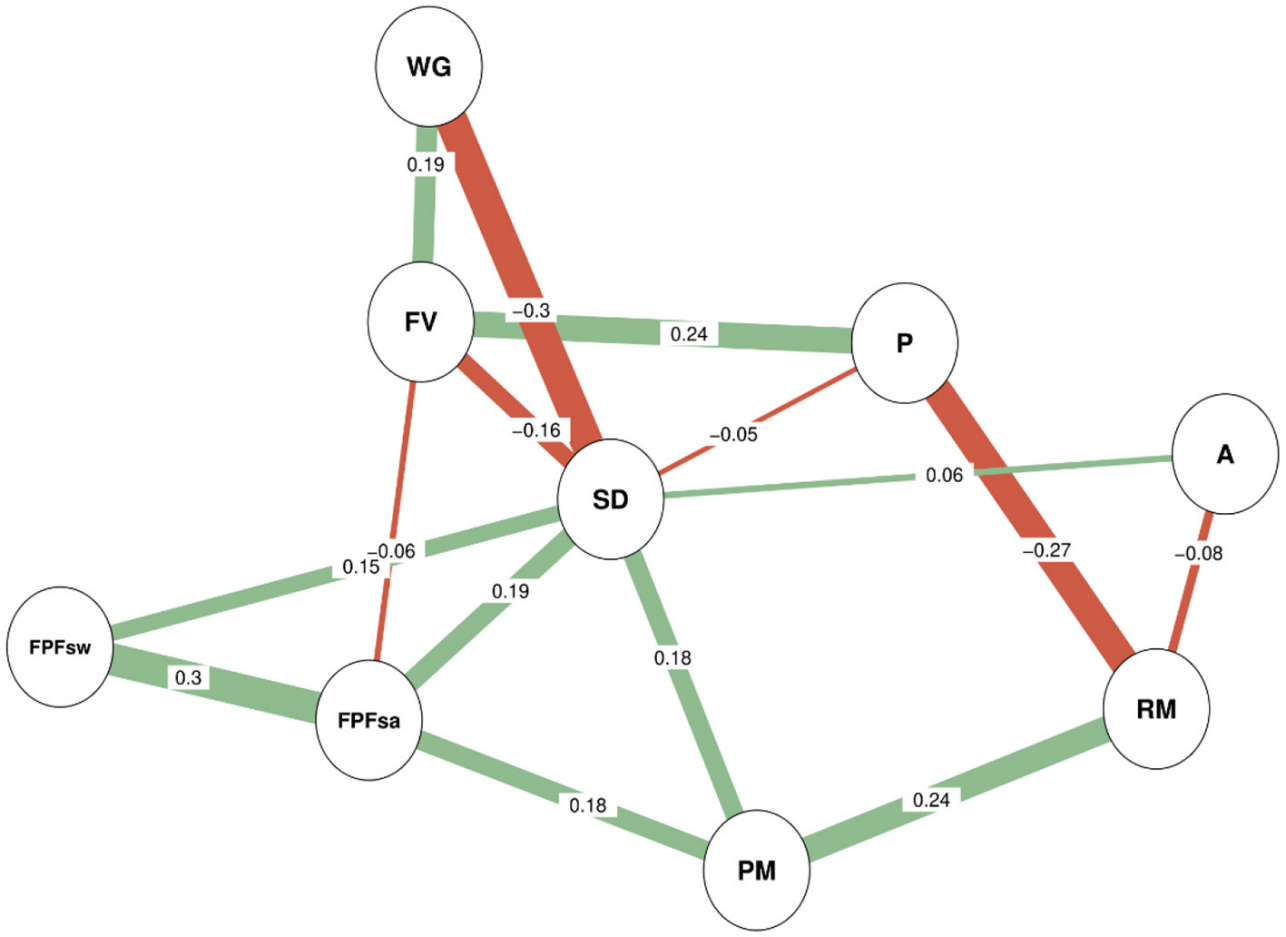
EBICglasso network model for the WCRF/AICR Screener. Estimated network model using only dietary recommendations (excluding BMI and physical activity). Green edges indicate positive partial correlations, and red edges indicate negative partial correlations between the variables, with edge thickness proportional to the magnitude of the relationship. A, Alcoholic drinks; FPFsa, Fast and processed food—savory; FPFsw, Fast and processed food—sweet; FV, fruit and vegetables; P, pulses; PM, processed meat; RM, red meat; SD, sugar-sweetened drinks; WG, wholegrains.

The initial network model incorporated the seven recommendations included in the Screener. Both “Body composition” and “Physical activity” appeared completely isolated, and without any significant connections to the other variables. Thus, a new network model was estimated by omitting them and focusing only on dietary recommendations. This decision was also based on conceptual considerations, as body composition and physical activity represent constructs that differ from dietary intake, with body composition potentially reflecting downstream consequences of diet and physical activity and physical activity representing a separate behavioral domain rather than a dietary behavior itself. Figure 2 displays the network model and the edge weights, which represent partial correlation coefficients, indicating relationships between variables after accounting for the influence of the rest.

The estimated network was comprised of nine variables with 16 non-zero edges out of 36 possible connections (connectivity ratio = 44.4%). Strong positive associations were observed between “Processed meat” and “Red meat” (0.24), “Processed meat” and “Fast and processed foods”—savory (0.18), “Fruit and vegetables” and “Wholegrains” (0.19), and between “Fast and processed foods”—sweet and “Fast and processed foods”—savory (0.30). Conversely, negative associations were found between “Red meat” and “Pulses” (−0.27), and among “Sugar-sweetened drinks” and “Wholegrains” (−0.30). The “Alcoholic drinks” variable appeared largely isolated from the main network and was only positively connected with “Sugar-sweetened drinks” (0.06) with an unexpected slightly negative relationship with “Red meat” (−0.08). Edge values and weights can be found in Supplementary Tables S7, S8.

**Figure 3:**
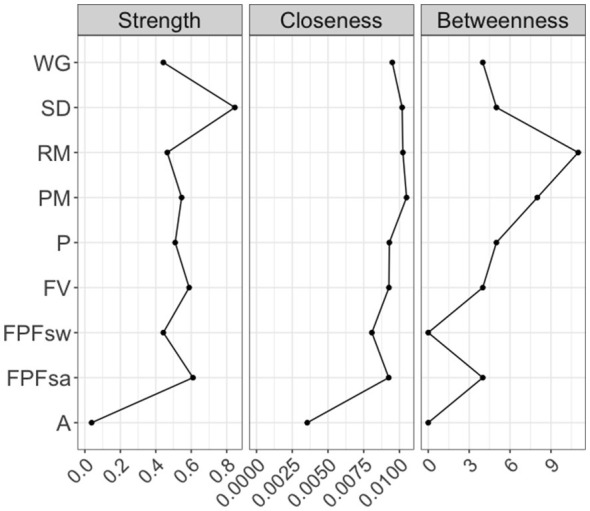
Centrality plot of the network displaying strength, closeness, and betweenness of the variables. Variable strength [range: 0.00–1.00], closeness [range: 0.00–0.02], and betweenness [range: 0.00–8.00]. A, alcoholic drinks; FPFsa, fast and processed food—savory; FPFsw, Fast and processed food—sweet; FV, fruit and vegetables; P, pulses; PM, processed meat; RM, red meat; SD, sugar-sweetened drinks; WG, wholegrains.

Regarding centrality analysis (Figure 3; Supplementary Table S9) “Sugar-sweetened drinks” exhibited the highest values across all measures (Strength: > 0.90, Closeness > 0.01, Betweenness ~ 7.00), suggesting its significant association with other dietary variables. On the other hand, “Alcoholic drinks” exhibited the lowest centrality across all measures (Strength ~ 0.20, Closeness < 0.07, Betweenness = 0.00), pointing to its relative isolation from the primary dietary network structure. “Fast and processed foods”—savory also showed relatively high strength centrality (>0.70), whereas “Processed meat” presented a high value in closeness centrality (~0.01), which, together with “Sugar-sweetened drinks” (< 0.01), indicates they have the shortest average path to other variables in the network. Finally, “Red meat” exhibited the highest betweenness centrality (~8.00), followed by “Sugar-sweetened drinks” (~7.00), “Processed meat” (~6.00) and “Pulses” (~4.00), indicating a potential bridging role in the network.

Among the network's structure-sample dependence, drop-case bootstrap stability analysis (Supplementary Figure S5) indicated that strength centrality showed the highest stability (correlation >0.70) with the original network when the number of retained participants was reduced to 54 (51.8% of the original sample). Closeness centrality demonstrated moderate stability, with correlations dropping below 0.70 with the same number of retained participants. Betweenness centrality exhibited the lowest stability, with correlations falling below 0.70 when fewer than 63 participants were retained (43.8% of the original sample).

Finally, our dietary network exhibited small-world properties, with an S. rand index of 1.11 and an S. HG index of 1.59.

## 5 Discussion

The WCRF/AICR Cancer Prevention Recommendations play a key role in guiding cancer prevention policies and strategies. The WCRF/AICR Screener translates these recommendations into a practical tool for rapid and valid assessment of nutritional and lifestyle factors relevant to cancer prevention and for identifying broad adherence patterns that may inform intervention strategies. In this study, the WCRF/AICR Screener showed moderate-to-strong correlations between overall scores when compared with validated methods, although agreement varied across individual recommendations. Network analysis provided an exploratory and preliminary characterization of the dietary pattern interrelationships in this population.

For validation purposes, we evaluated agreement and consistency between the WCRF/AICR Screener and validated, objective measurements. The Screener showed moderate-to-strong correlation with two validated reference scores, similar to previously reported value in other lifestyle screener validation study (32). Furthermore, regression analysis using both validated methods indicated the WCRF/AICR Screener can reliably rank individuals according to their adherence to cancer prevention recommendations. The high correlation values obtained in the test-retest analysis also indicates strong consistency and reliability of the Screener, as previously published (8). Absolute agreement between the Screener and the validated tools was stronger when the comparison was made using data obtained through the EMDA-score, which is consistent with extensive evidence showing systematic overestimation of food intake in FFQs when compared with weighed food records or 24-h recalls, explaining the wider limits of agreement. However, proportional bias could suggest that extreme values may not be accurately captured by the WCRF/AICR Screener in this population, indicating a systematic overestimation of adherence among participants with lower scores and underestimation in those with higher adherence. These results suggest caution when interpreting extreme scores.

Regarding recommendation-specific agreement, results varied according to the reference method used and were heterogeneous across individual recommendations. Although both reference methods are validated dietary assessment approaches, they capture dietary intake in different ways and may be affected by different sources of measurement error. Moderate-to-high agreement was observed, particularly for “Body composition” and “Plant-based foods”, whereas “Physical activity” and “Red and processed meat” showed lower concordance. Some components, such as “Pulses” and “Wholegrains”, showed low or null kappa values when the Screener was compared with *Validated Score-EMDA*, despite moderate cross-classification percentages. However, this pattern was not consistently observed when using *Validated Score-FFQ*, for which agreement improved for several components, including “Pulses”, “Wholegrains”, and “Red and processed meat”. Although both reference scores were based on validated assessment methods, the *Validated Score-FFQ* may be methodologically closer to the Screener, as both approaches rely on reported frequencies of habitual consumption of different food groups. This may partly explain the higher recommendation-specific agreement observed with the *Validated Score-FFQ* compared with the *Validated Score-EMDA*. These findings indicate that, although the Screener appears useful for ranking overall adherence, caution is warranted when interpreting recommendation-specific scores.

The apparent discrepancy between moderate cross-classification percentages and low or null weighted kappa values may partly reflect the distribution of participants across score categories. For some recommendations, particularly “Pulses” and “Wholegrains”, most participants were concentrated in a single category according to the validated method, which can reduce kappa values despite apparently acceptable crude agreement. Therefore, cross-classification percentages and kappa coefficients should be interpreted jointly, as they capture complementary aspects of agreement. A comparable low agreement aligns with the one observed for “Red meat” in the EMDA validation (33), where animal protein (including red meat) showed poorer agreement. Finally, low agreement observed for “Physical activity” may reflect desirability bias, whereby participants increase their activity levels, known as the. Hawthorne effect (34, 35).

The exploratory network revealed non-random patterns of dietary intake. Bootstrap resampling suggested robust overall structure, though the weakest associations were less stable. In contrast, the strongest associations showed high stability, and therefore greater reliability. Preliminary food group clustering showed proximity between healthier foods and, separately, less healthy ones. “Processed meat” clustered with “Red meat”, while “Fast and processed foods”—savory connected to “Processed meat” and to “Fast and processed foods”—sweet. Similar clustering of processed meats, fast foods, and sugary items has been observed in network-based dietary studies (36), and reinforce the potential utility of network models in capturing co-occurrence dietary patterns, likely reflecting socioeconomic or behavioral influences (37, 38).

Centrality analyses indicated a preliminary understanding of variable positioning within the network. “Sugar-sweetened drinks” and “Fast and processed foods”—savory had the highest strength centrality, showing stronger conditional associations with other components. In terms of closeness, “Sugar-sweetened drinks” and “Processed meat” showed the shortest average distance to other variables, suggesting that their consumption could be frequently associated with other food groups. This interpretation should however be interpreted with caution given its lower stability in the bootstrap analysis. Prior research beyond dietary network analysis has shown that sugar-sweetened beverages can influence the consumption of other food groups (39) and are associated with increased risk of overweight and obesity (40). Their high centrality in our network analysis generates the hypothesis that they may occupy an important position within unhealthy dietary profiles in this population, but this finding should not be interpreted as evidence of a causal or intervention effect.

In addition, identifying small-world properties supports the exploratory hypothesis that certain central food items, such as “Sugar-sweetened drinks”, could influence broader dietary patterns due to their central position and interconnectedness. Nevertheless, given the small sample size and limited stability of betweenness centrality, these results remain exploratory and require confirmation in larger and more heterogeneous samples before being used to guide intervention strategies. Importantly, “Alcoholic drinks” appeared largely isolated, consistent with prior research indicating that in young adults, alcohol consumption often follows social and behavioral trajectories independent from dietary habits (37, 41, 42).

Several limitations should be acknowledged. First, the sample, recruited though convenience sampling in a single university setting, comprised mostly of healthy, educated individuals with a healthy BMI and in good physical form, which limits the generalizability to other young adult populations (43). Therefore, the findings may not be generalizable to broader young adult populations with more diverse socioeconomic, educational, and health backgrounds. In line with this, even participants in the lowest adherence group showed relatively low body fat (44). In contrast, unlike studies that stratify by clinical conditions (45, 46), our work focused on co-occurrence patterns within a relatively healthy population. Additionally, although EMDA reduces reporting bias, it remains a self-reported measure and is therefore subjected to potential misreporting and social desirability bias (47). Finally, while simulation studies suggest that network analysis provide more stable estimates when the number of nodes is limited (14), and recommend bootstrap resampling to assess robustness, the modest sample size (*n* = 112) warrants interpreting the present results as exploratory. Studies with larger and more heterogeneous participants are needed to confirm the observed network patterns.

At the same time, the study presents several strengths. The integration of the EMDA with the FFQ data enhances reliability and helps mitigate method-specific biases. Additionally, using short 2-hR periods, rather than traditional 24-hR, enables near real-time reporting, reduces recall bias and improves accuracy, particularly for contextual details such as whether foods were homemade, ready-made, or take-away. This was essential for capturing fast and processed food consumption. The detailed characterization of the population further strengthens the analyses, and the use of dietary network approaches provides insights into lifestyle patterns which may inform contextually relevant interventions and generate new research hypothesis. Future studies in more diverse populations will enhance its utility in preventive healthcare.

## 6 Conclusion

The WCRF/AICR Screener proved reliable in ranking young individuals in a healthy, university-based sample according to their adherence to cancer prevention guidelines. Network analysis provided exploratory insights into dietary patterns, consistently identifying intake of sugar-sweetened drinks as a central and highly connected variable across all centrality measures. This could potentially imply that sugar-sweetened drinks may be embedded within broader dietary behavior patterns in similar young adult populations. In contrast, alcoholic intake appeared relatively isolated, indicating it could play a limited role in shaping the overall dietary structure. Future research should explore these patterns with more diverse socioeconomic, educational, and health backgrounds, as well as consider longitudinal designs to assess temporal changes and confirm whether highly connected variables have practical relevance for intervention designs.

## Data Availability

The datasets used and/or analyzed during the current study are available from the corresponding author on reasonable request. Requests to access the datasets should be directed to Dora Romaguera, mariaadoracion.romaguera@idisba.
