# Supplementary material for: Validity, consistency and network structure of the World Cancer Research Fund/American Institute for Cancer Research Screener in young adults
Source: Front Nutr. 2026 Jul 16;13:1764935. doi: 10.3389/fnut.2026.1764935 (PMC13421909; doi:10.3389/fnut.2026.1764935)
Supplement: Supplementary file 1 [file Supplementary_file_1.docx]

**Summary of the file names and types**

**Supplementary Table 1.** Breakdown of the WCRF/AICR Screener in English.

**Supplementary Table 2.** Breakdown of the WCRF/AICR Screener in Spanish

**Supplementary Table 3.** Distribution of food items from the FFQ into the WCRF/AICR Screener categories

**Supplementary Table 4.** Variables included in the *a priori* network estimation.

**Supplementary Table 5.** Dietary characteristics of the validation study population according to tertiles of the WCRF/AICR Screener

**Supplementary Table 6.** Distribution and agreement between scores of each WCRF/AICR recommendation via Screener and *Validated Score-FFQ* (n=101)

**Supplementary Table 7.** Matrix with all the networks’ partial connections between variables

**Supplementary Table 8.** Network edge weights with bootstrapped confidence intervals

**Supplementary Table 9.** Centrality indices and stability coefficients of the dietary network

**Supplementary Figure 1.** Study scheme

**Supplementary Figure 2.** Flowchart of the study

**Supplementary Figure 3.** Bland-Altman Plot for the agreement of the scores derived from the WCRF/AICR Screener and *Validated Score-EMDA*

**Supplementary Figure 4.** Bland-Altman Plot for the agreement of the scores derived from the WCRF/AICR Screener and *Validated Score-FFQ*

**Supplementary Figure 5.** Edge weight stability analysis in the network: comparison of sample estimates and bootstrap results

**Supplementary Table 1.** Breakdown of the WCRF/AICR Screener in English

| **QUESTION** | | **ANSWER** | **SCORING** |
| --- | --- | --- | --- |
| **SECTION 1: BODY COMPOSITION** | | | |
| **What is your current weight and height?**  If available, measure your weight and height on the same day of the screener. If this is not possible, use a measurement that you remember.  In order to weigh yourself, step on a scale without shoes and with light or no clothes. Measure your height without shoes also, keeping feet flat and a straight back. | | **Weight:** _________ kg  **Height:** _________ cm | Body mass index (BMI) is calculated automatically as weight in kg divided by squared height in m (kg/m^2^).  **BMI 18.5-24.9 kg/m^2^ 1**  **BMI 25-29.9 kg/m^2^ 0.5**  **BMI <18.5 or ≥30 kg/m^2^ 0** |
| **What is your current waist circumference?**    If available, measure waist circumference on the same day of the screener (by yourself or by a health professional). If this is not possible, please indicate “I don’t know my current waist circumference”.    In order to measure your waist, stand up, take off bulky clothing and place your feet shoulder-with apart. Wrap a tape measure around the widest part of your belly, roughly in line with your belly button. Make sure it is resting directly against your skin and you are breathing normally. | | **Waist circumference (WC)**: _____ cm  How was the measurement obtained?   - Measured by a healthcare professional - Self-measured (right now) - I don´t know my current waist circumference | Males  **WC: <94 cm 1**  **WC: 94–<102 cm 0.5**  **WC: ≥102 cm 0**  Females  **WC: <80 cm 1**  **WC: 80–<88 cm 0.5**  **WC: ≥88 cm 0** |
| An overall score for body composition is calculated as an average: score (BMI) + score (WC) / 2. If a person chooses the option “I don’t know my current waist circumference”, the score is based only on their BMI. | | | |
| **SECTION 2: PHYSICAL ACTIVITY** | | | |
| **Do you carry out light physical activity on a regular basis?** *Examples: Light walking, fishing, light housework and stretching.* | | - **YES** - **NO** | MVPA = (Min/day*days/week of MPA) + (Min/day*days/week of VPA*2).  **>=150min/week 1**  **>=75 - <150min/week 0.5**  **<75min/week of MVPA 0** |
| **How many days a week do you carry out light physical activity?** | | _________Days/week |  |
| **On those days, how many minutes a day do you do light physical activity?** | | _________Min/day |  |
| **Do you carry out moderate physical activity on a regular basis?**  When carrying out moderate physical activity, you can talk, but cannot sing. *Examples: Fast walking, dancing, riding a bike, gardening, hiking, general construction work, hunting, food collection, cleaning. Also consider daily activities, such as cycling or walking to work.* | | - **YES** - **NO** |  |
| **How many days a week do you carry out moderate physical activity?** | | _________Days/week |  |
| **On those days, how many minutes a day do you do moderate physical activity?** | | _________Min/day |  |
| **Do you carry out vigorous physical activity on a regular basis?**  When carrying out vigorous physical activity, you are not able to talk more than 4-5 words. *Examples: Jogging, fast swimming, aerobics, competitive sports, heavy weightlifting, intense digging or lifting heavy objects in the workplace* | | - **YES** - **NO** |  |
| **How many days a week do you carry out vigorous physical activity?** | | _________Days/week |  |
| **On those days, how many minutes a day do you do vigorous physical activity?** | | _________Min/day |  |
| Only moderate physical activity (MPA) and vigorous physical activity (VPA) is included in the calculations to score physical activity. | | | |
| **SECTION 3. DIET**  In the following section, we would like you to indicate how often you eat a variety of food items. Please use the examples provided as a guide. | | | |
| **SECTION 3.1. PLANT-BASED FOODS** | | | |
| **How many portions of fruit and vegetables do you eat a day?**  Add up all the portions of fruits and non-starchy vegetables you eat in a day.  A portion of fruit is equivalent to:   - 1 apple, pear, orange, banana, peach, kiwi; a slice of melon or watermelon. - 2-3 clementines, figs, plums, apricots. - 1 handful of strawberries, grapes, cherries.   A portion of non-starchy vegetables is equivalent to:   - A big bowl of salad. - A bowl of vegetable soup, minestrone. - A small plate of cooked vegetables. - ½ aubergine, courgette; 1 medium tomato; 1 big carrot.   Do not count potatoes, sweet potatoes or yucca. | - 5 portions or more/day - 4 portions/day - 2-3 portions/day - 1 portion/day - Less than 1 portion/day - I don’t usually eat fruit and vegetables | | **5 portions or more/day 1**  **2-4 portions/day 0.5**  **≤1 portions/day 0** |
| **How many portions of pulses do you eat a week?**  A portion of pulses is equivalent to:   - A bowl of lentils, chickpeas, pinto, black or white beans, peas. - A legume salad. | - 4 portions or more/week - 3 portions/week - 2 portions/week - 1 portion/week - Less than 1 portion/week - I don’t usually eat pulses | | **3 portions or more/week 1**  **1-2 portions/week 0.5**  **<1 portions/week 0** |
| **How often do you eat wholegrains?**  Examples of wholegrain foods and servings are:   - 2 slices of wholegrain bread. - 1 plate of cooked wholegrain pasta, cooked brown rice, quinoa. - 1 small bowl of wholegrain low-sugar breakfast cereals (cooked oatmeal, porridge). | - Multiple times/day - Once a day - 4-6 times/week - 1-3 times/week - Less than once a week - I don’t usually eat wholegrains | | **>1 portions/day 1**  **1 portion/day 0.5**  **<1 portion/day 0** |
| An overall score for fruit & vegetables, pulses and wholegrains is calculated as an average:  Score (fruit & vegetables) + (pulses) + (wholegrains) / divided by 3 | | | |
| **SECTION 3.2. FAST FOODS AND PROCESSED FOODS HIGH IN FAT, STARCHES OR SUGARS** | | | |
| **How often do you eat fast or processed foods and snacks?**  Examples of fast and processed foods are:   - Salty snacks: crisps, salty nuts or crackers. - Pre-prepared, frozen dishes or takeaways: pizza, lasagne, nuggets, croquettes, chips. - Mayonnaise, ketchup, *tomate frito*, mustard, cream cheese | - 4 times or more/week - 3 times/week - Twice/week - Once a week - Less than once a week* - I don’t usually eat fast or processed food | | **≤1 times/week 1**  **2-3 times/week 0.5**  **≥ 4 times/week 0** |
| **How often do you eat sweets and pastries?**  Examples of sweets and pastries are:   - Biscuits, doughnuts, croissants, cakes. - Chocolate bars, sweets. - Ice-cream, desserts (flan, *crème brûlée*, pudding, *fromage frais*). - Sugary breakfast cereals. | - 4 times or more/week - 3 times/week - Twice/week - Once a week - Less than once a week* - I don’t usually eat fast or processed food | |  |
| An overall score for fast foods and processed foods is calculated adding up: (times/week of fast or processed foods) + (times/week of sweets & pastries).  *Less than once a week is considered 0.5 times | | | |
| **SECTION 3.3. RED AND PROCESSED MEATS** | | | |
| **How many portions of red meat do you eat a week?**  Examples of red meats are beef, pork, lamb, game meat and offal.  A portion of cooked red meat is equivalent to:   - 1 steak the size of the palm of your hand. - 2-3 lamb or pork chops. - Minced meat (butcher) 2-3 meatballs, bolognaise. - 1 serving of liver or kidney. | - 6 portions or more/week - 4-5 portions/week - 3 portions/week - 1-2 portions/week - Less than 1 portion/week - I don’t usually eat red meat | | **≤3 portions/week red meat**  **& <1 portion/week**  **processed meat 1**  **≤3 portions/week red meat &**  **1/week processed meat 0.5**  **>3 portions/week red meat**  **or ≥2 portions/week**  **processed meat 0** |
| **How often do you eat processed meat?**   - 2-3 slices of ham, bacon, turkey*.* - 1 sausage, 1 hotdog, 1 hamburger (not homemade). - 1 can of foie-gras. | - 4 portions or more/week - 3 portions/week - 2 portions/week - 1 portion/week - Less than 1 portion/week - I don’t usually eat processed meat | |  |
| **SECTION 3.4. SUGAR-SWEETENED DRINKS** | | | |
| **How often do you have sugary drinks?**  A portion is equivalent to 1 glass (250 ml). Examples of sugary drinks include:   - Non-diet soft drinks (cola, tonic, iced tea). - Energy drinks, sports drinks, sweetened waters. - Cocoa-based drinks or similar store-bought, sugary milkshakes (vanilla, strawberry, etc.). - Store-bought, pre-prepared sugary coffee and tea (*Starbucks*). - Fruit drinks and juices with added sugars and nectars. | - 1 drink or more/day - 4-6 drinks/week - 2-3 drinks/week - 1 drink/week - Less than 1 drink/week - I don’t usually have sugary drinks | | **No sugary drinks 1**  **<1-6 drinks/week 0.5**  **≥1 drinks/day 0** |
| **SECTION 3.5. ALCOHOLIC DRINKS** | | | |
| **How often do you have an alcoholic drink?**  Examples of alcoholic drinks include:   - 1 small glass of wine (125 ml). - 1 can or bottle of beer (330 ml). - 1 shot of spirits or liqueur (25 ml) on its own or combined with a soft drink. Please note that a double shot (50 ml) counts as 2 drinks. - 1 small glass of champagne (125 ml). | Males   - More than 2 drinks/day - 1-2 drinks/day - 4-6 drinks/week - 1-3 drinks/week - Less than 1 drink/week - I don’t usually have alcoholic drinks   Females   - More than 1 drink/day - 1 drink/day - 4-6 drinks/week - 1-3 drinks/week - Less than 1 drink/week - I don’t usually have alcoholic drinks | | Males  **No alcoholic drinks 1**  **<1 to 2 drinks/day 0.5**  **>2 drinks/day 0**  Females  **No alcoholic drinks 1**  **<1 to 1 drink/day 0.5**  **>1 drink/day 0** |
| **TOTAL SCORE:** SECTION 1 + SECTION 2 + SECTION 3.1 + SECTION 3.2 + SECTION 3.3 + SECTION 3.4 + SECTION 3.5  Score range: [0-7] | | | |

**Supplementary Table 2.** Breakdown of the WCRF/AICR Screener in Spanish

| **PREGUNTA** | | **RESPUESTAS** | **PUNTUACIÓN** |
| --- | --- | --- | --- |
| **SECCIÓN 1: MEDIDAS CORPORALES** | | | |
| **¿Cuáles son su peso y altura actuales?**  Si es posible, mida su peso y talla el mismo día de la evaluación. Si no es posible, use una medida auto-reportada (que recuerde del último año). Para medir su peso, debe usar una balanza sin zapatos y con ropa ligera o sin ropa. Para medir su altura, recuerde no llevar zapatos y tener la espalda recta. | | **Peso:** _________ kg  **Altura:** _________ cm | El índice de Masa Corporal (IMC) se debe calcular como peso en kg dividido por el cuadrado de la altura en metros (kg/m2).  **IMC 18.5-24.9 kg/m^2^ 1**  **IMC 25-29.9 kg/m^2^ 0.5**  **IMC <18.5 o ≥30 kg/m^2^ 0** |
| **¿Cuál es su circunferencia de la cintura actual?**  Si es posible, mida su cintura el mismo día de la evaluación (por usted mismo o por un profesional sanitario). Si no es posible, marque la opción NS/NC.  La circunferencia de la cintura se debe medir con una cinta métrica flexible a la altura del ombligo, procurando que la cinta métrica quede recta y sin ropa entre la cinta y la piel. La posición corporal debe ser relajada, espirando con normalidad, de pie y con los pies separados a la anchura de los hombros. | | **Circunferencia de la cintura (CC):** _________ cm  ¿Cómo se obtuvo la medida?   - - Medido por personal sanitario   - Auto-medido (ahora mismo)   - NS/NC * | Hombres:  **CC: <94 cm 1**  **CC: 94–<102 cm 0.5**  **CC: ≥102 cm 0**  Mujeres:  **CC: <80 cm 1**  **CC: 80–<88 cm 0.5**  **CC: ≥88 cm 0** |
| A la puntuación general de la composición corporal se calcula como un promedio: puntuación (IMC) + puntuación (CC) / 2; * Si una persona elige la opción “NS/NC” su CC, la puntuación se basará únicamente en su IMC. | | | |
| **SECCIÓN 2: ACTIVIDAD FÍSICA** | | | |
| **¿Realiza actividad física ligera de forma habitual?**  *Ejemplos: Caminar despacio (paseo), pescar, tareas del hogar ligeras, estiramientos ligeros.* | | - **SÍ** - **NO** | AFMV= (Min/día*días/semana de AFM) + (Min/día*días/semana de AFV*2).    **>=150min/semana 1**  **>=75 - <150min/semana 0.5**  **<75min/semana 0** |
| **¿Cuántos días a la semana hace actividad física ligera de promedio?** | | _________Días/semana |  |
| **Durante esos días, ¿cuántos minutos al día hace actividad física ligera?** | | _________Min/día |  |
| **¿Realiza actividad física moderada (AFM) de forma habitual?**  Durante la realización de actividad física moderada, usted puede hablar, pero no cantar.  *Ejemplos: Caminar rápido, bailar, montar en bicicleta, jardinería, excursiones, recolecta de alimentos, actividades de limpieza. Considere también trayectos del día a día, como ir al trabajo en bici o caminando.* | | - **SÍ** - **NO** |  |
| **¿Cuántos días a la semana hace actividad física moderada de promedio?** | | _________Días/semana |  |
| **Durante esos días, ¿cuántos minutos al día hace actividad física moderada?** | | _________Min/día |  |
| **¿Realiza actividad física vigorosa de forma habitual?**  Durante la realización de actividad física vigorosa, usted no puede hablar más de 4 o 5 palabras. *Ejemplos: Correr, natación, aerobic, deportes de competición, levantar pesas, trabajos de construcción como perforar, o levantar objetos pesados en el lugar de trabajo.* | | - **SÍ** - **NO** |  |
| **¿Cuántos días a la semana hace actividad física vigorosa de promedio?** | | _________Días/semana |  |
| **Durante esos días, ¿cuántos minutos al día hace actividad física vigorosa?** | | _________Min/día |  |
| En los cálculos para puntuar la actividad física sólo se incluyen la actividad física moderada (AFM) y la actividad física vigorosa (AFV). | | | |
| **SECCIÓN 3. DIETA**  En la siguiente sección, nos gustaría que indicara la frecuencia con la que consume diversos alimentos. Use los ejemplos proporcionados como guía. | | | |
| **SECCIÓN 3.1. ALIMENTOS DE ORIGEN VEGETAL** | | | |
| **Sume todas las porciones de fruta y verdura que consume en un día.**  Una porción de fruta equivale a:   - 1 manzana, pera, naranja, plátano, melocotón, kiwi; 1 tajada de melón o sandía. - 2-3 clementinas, higos, ciruelas, albaricoques. - 1 puñado de fresas, uvas, cerezas.   Una porción de verdura equivale a:   - 1 plato grande de lechuga. - 1 plato hondo de puré de verdura, gazpacho. - 1 plato pequeño de verduras cocinadas. - ½ berenjena o calabacín; 1 tomate mediano; 1 zanahoria grande.   No cuente como verdura las patatas, boniatos o yuca. | - 5 porciones o más/día - 4 porciones/día - 2-3 porciones/día - 1 porción/día - Menos de 1 porción/día - No suelo consumir fruta y verdura | | **≥** **5 porciones /día 1**  **2-4 porciones /día 0.5**  **≤ 1 porciones /día  0** |
| **¿Cuántas porciones de legumbres consume a la semana?**   - Una porción de legumbres equivale a: - 1 plato hondo de lentejas, garbanzos, alubias pintas, negras o blancas, guisantes. - 1 ensalada de legumbres. | - 4 porciones o más/semana - 3 porciones/semana - 2 porciones/semana - 1 porción/semana - Menos de 1 porción/semana - No suelo consumir legumbres | | **≥ 3 porciones/semana 1**  **1-2 porciones/semana 0.5**  **<1 porciones/semana 0** |
| **¿Con qué frecuencia consume cereales y alimentos integrales?**  Algunos ejemplos de cereales y productos integrales incluyen:   - 2 rebanadas de pan integral. - 1 plato de pasta integral cocida, arroz integral cocido, y/o quinoa. - 1 bol pequeño de cereales de desayuno integrales bajos en azúcar (avena cocida/porridge). | - Varias veces/día - 1 vez/día - 4-6 veces/semana - 1-3 veces/semana - Menos de 1 vez/semana - No suelo consumir alimentos integrales | | **>1 porción/día 1**  **1 porción/día 0.5**  **<1 porción/día 0** |
| La puntuación global de frutas y verduras, legumbres y productos integrales se calcula como media:  Puntuación (frutas y hortalizas) + (legumbres) + (cereales integrales) / dividido por 3 | | | |
| **SECCIÓN 3.2. COMIDA RÁPIDA, PROCESADA, REPOSTERIA INDUSTRIAL** | | | |
| **¿Con qué frecuencia consume comida rápida, procesada o aperitivos salados?**  Algunos ejemplos de comida rápida y alimentos procesados son:   - Aperitivos salados y fritos: patatas fritas de bolsa, frutos secos salados y/o fritos, gusanitos. - Platos preparados y/o congelados, o para llevar: pizza, lasaña, nuggets, croquetas, patatas fritas. Mayonesa, kétchup, salsa de tomate frito, mostaza, quesos untables. | - 4 veces o más/semana - 3 veces/semana - 2 veces/semana - 1 vez/semana - Menos de 1 vez/semana * - No suelo consumir alimentos procesados | | **≤1 vez/semana 1**  **2-3 veces/semana 0.5**  **≥ 4 veces/semana 0** |
| **¿Con qué frecuencia consume repostería industrial y otros dulces procesados?**  Algunos ejemplos de repostería industria y dulces procesados son:   - Dulces y repostería industrial: galletas, donuts, cruasanes, chocolatinas, caramelos, tartas, bizcocho. - Postres (flan, natillas, pudding, Petit Suisse), helados. - Cereales de desayuno azucarados. | - 4 veces o más/semana - 3 veces/semana - 2 veces/semana - 1 vez/semana - Menos de 1 vez/semana * - No suelo consumir alimentos dulces procesados | |  |
| Se calcula una puntuación global para comidas rápidas y procesadas sumando: veces/semana de (comidas rápidas o procesadas) + (repostería industrial y dulces procesados); *Menos de una vez a la semana se considera 0,5 veces. | | | |
| **SECCIÓN 3.3. CARNE ROJA Y PROCESADA** | | | |
| **¿Cuántas porciones de carne roja consume a la semana?**  La carne roja hace referencia a la carne de ternera, cerdo, cordero, carne de caza y vísceras.   - Una porción de carne roja cocinada equivale a: - 1 bistec del tamaño de la palma de tu mano. - 2-3 chuletas de cordero o cerdo. - Carne picada (carnicería): 2-3 albóndigas, boloñesa. - Ración de hígado o riñón. | - 6 porciones o más/semana - 4-5 porciones/semana - 3 porciones/semana - 1-2 porciones/semana - Menos de 1 porción/semana - No suelo consumir carne roja | | **≤3 raciones/semana  de carne roja y  <1 ración/semana de  carne procesada 1**  **≤3 porciones/semana  de carne roja y 1/semana  de carne procesada 0.5**  **>3 porciones/semana  de carne roja o  ≥2 porciones/semana  de carne procesada 0** |
| **¿Cuántas porciones de carne procesada consume a la semana?**   - Una porción de carne procesada equivale a: - 2-3 lonchas de jamón cocido, jamón serrano, chorizo, sobrasada, salchichón, fuet, caña de lomo, morcilla, beicon, pavo. - 1 salchicha, 1 Frankfurt, 1 hamburguesa no casera. - 1 lata de fuagrás. | - 4 porciones o más/semana - 3 porciones/semana - 2 porciones/semana - 1 porciones/semana - Menos de 1 porción/semana - No suelo consumir carne procesada | |  |
| **SECCIÓN 3.4. BEBIDAS AZUCARADAS** | | | |
| **¿Cuántas bebidas azucaradas consume?**  Una porción de bebida azucarada equivale a 1 vaso (250 ml) de:   - Refrescos azucarados (sabor cola, naranja, limón, tónica, té frío). - Bebidas energéticas, bebidas deportivas, aguas azucaradas. - Bebidas de cacao (Cola-Cao, Laccao) o batidos de leche azucarados industriales (vainilla, fresa, etc.). - Café y té preparado, azucarado o industrial (Starbucks). - Bebidas de fruta con azúcar añadido y néctares. | - 1 bebida o más/día - 4-6 bebidas/semana - 2-3 bebidas/semana - 1 bebida/semana - Menos de 1 bebida/semana - No suelo consumir bebidas azucaradas | | **Sin bebidas azucarada 1**  **<1-6 bebidas/semana 0.5**  **≥ 1 bebida/día 0** |
| **SECCIÓN 3.5. BEBIDAS CON ALCOHOL** | | | |
| **¿Cuántas bebidas alcohólicas consume?**  Una bebida alcohólica equivale a:   - 1 copa de vino (125 ml). - 1 lata o botella de cerveza (330 ml). - 1 chupito de licor (25 ml), solo o combinado con refresco. Tenga en cuenta que los combinados llevan 50 ml de licor y por tanto cuentan como 2 bebidas. - 1 copa de cava (125 ml). | **Masculino:**   - Más de 2 bebidas/día - 1-2 bebidas/día - 4-6 bebidas/semana - 1-3 bebidas/semana - Menos de 1 bebida/semana - No suelo consumir bebidas alcohólicas   **Femenino:**   - Más de 1 bebida/día - 1 bebida/día - 4-6 bebidas/semana - 1-3 bebidas/semana - Menos de 1 bebida/semana - No suelo consumir bebidas alcohólicas | | Hombres  **0 bebidas alcohólicas 1**  **<1 a 2 bebidas/día 0.5**  **>2 bebidas/día 0**    Mujeres  **0 bebidas alcohólicas 1**  **<1 a 1 bebidas/día 0.5**  **>1 bebida/día 0** |
| **Puntuación total: APARTADO 1 + APARTADO 2 + [(APARTADO 3.1 + APARTADO 3.2 + APARTADO 3.3)/3] + APARTADO 3.4 + APARTADO 3.5**  **Escala de puntuación: [0-7]** | | | |

**Supplementary Table 3.** Distribution of food items from the FFQ into the WCRF/AICR Screener categories

| **Fruits and vegetables** | Chard, spinach, cabbage, cauliflower, broccoli, brussels sprouts, lettuce, chicory, endive, tomato, carrot, pumpkin, green beans, canned corn, eggplant, zucchini, cucumbers, peppers, asparagus, gazpacho, onion, garlic, mushrooms, vegetable puree, orange, mandarin orange, grapefruit, banana, apple, pear, strawberries, berries (blueberries, blackberries or raspberries), peach, apricot, nectarine, cherries, plums, watermelon, melon, grapes, pineapple, avocados, mango, papaya, persimmon, kiwi. |
| --- | --- |
| **Pulses** | Lentils, chickpeas, kidney beans, dry soybeans, peas, beans. |
| **Wholegrains** | Whole wheat loaf, *pan moreno* (semi wholegrain bread), wholegrain bread sticks, wholegrain cereals (muesli without added sugar, rolled oats), All-Bran, brown rice, wholegrain pasta, wholegrain couscous, quinoa. |
| **Fast and processed foods (Savoury)** | Cheese wedges, cream cheese, cheese slices for melting, fast foods (store-bought burger, pizza, kebab, fried chicken), soups, broths and creams in sachets or cartons, pre-cooked foods (croquettes, pizza, pies, nuggets, French fries), *tomate frito*, ketchup, mustard, mayonnaise, fried and/or salted nuts (peanuts, almonds, pistachios, cashew nuts, sunflower seeds), crisps, snacks. |
| **Fast and processed foods (Sweet)** | Sweet dairy or vegetarian dessert, custard, pudding, ice cream, sweetened breakfast cereals, cereal bars, biscuits, chocolate chip biscuits, muffins, *ensaïmada* (pastry)*,* croissant, donut and other industrial pastries, cakes, churros, *porras* and similar, chocolate, cocoa powder, nougat, shortbread, marzipan, candies, spreadable chocolate or similar, wholegrain biscuits |
| **Red meat** | Beef or veal, pork, lamb meat, liver (beef, pork, chicken) other offal, game meat (horse, wild boar) |
| **Processed meat** | Hamburger, ready-made meatballs, *serrano* ham, cooked ham, turkey cold cuts, processed meats and sausages (sausages, *salami, chorizo*, etc.), pate, *foie gras*, bacon. |
| **Sugar-sweetened drinks** | Packaged and/or sweetened milkshakes, carbonated beverages containing sugar (cola, lemonade, tonic or other drinks), energy drinks, sports drinks, sweetened waters, bottled or canned fruit juices, sweetened or industrial coffee and tea. |
| **Alcoholic drinks** | Beer, liqueurs, distilled spirits, red wine, white wine, rosé wine, sweet wines, cava. |

**Supplementary Table 4.** Variables included in the *a priori* network estimation.

| “BMI” (included in the network representing the variable “Body composition”), “Physical activity” and intake of “Fruit and vegetables”, “Pulses”, “Wholegrains”, “Fast and processed foods” - savoury and sweet, “Red meat”, “Processed meat”, “Sugar-sweetened drinks” and “Alcoholic drinks”. |
| --- |

Although WC was measured, it was not incorporated into the network analysis. The main reason for excluding this variable is the strong correlation between the variables “WC” and “BMI”, as both serve as indicators of adiposity. BMI: Body Mass Index; WC: Waist Circumference.

**Supplementary Table 5.** Dietary characteristics of the validation study population according to tertiles of the WCRF/AICR Screener

| **FFQ** | **Overall** | **T1**  **Low adherence** | **T2**  **Medium adherence** | **T3**  **High adherence** | *p value* |
| --- | --- | --- | --- | --- | --- |
| Participants, n | 112 | 38 | 37 | 37 |  |
| *Servings/day* | | | | | |
| Fruits & vegetables | 5.8 (2.6) | 5.2 (2.8) | 5.3 (2.7) | 6.8 (2.1) | 0.012 |
| Pulses | 0.5 (0.4) | 0.3 (0.2) | 0.4 (0.5) | 0.6 (0.5) | 0.006 |
| Wholegrains | 1.4 (0.9) | 1.1 (0.8) | 1.4 (1.1) | 1.8 (0.8) | 0.005 |
| FPF (savoury) | 0.9 (0.7) | 1.3 (0.9) | 1.0 (0.7) | 0.5 (0.4) | <0.001 |
| FPF (sweet) | 1.1 (1.0) | 1.7 (1.2) | 0.9 (0.8) | 0.7 (0.7) | <0.001 |
| Red meat | 0.3 (0.3) | 0.4 (0.3) | 0.3 (0.3) | 0.2 (0.2) | 0.037 |
| Processed meat | 0.7 (0.6) | 0.9 (0.6) | 0.7 (0.6) | 0.4 (0.5) | <0.001 |
| Sugar-sweetened drinks | 0.3 (0.3) | 0.3 (0.4) | 0.3 (0.3) | 0.1 (0.3) | <0.001 |
| Alcoholic drinks | 0.3 (0.4) | 0.3 (0.4) | 0.2 (0.2) | 0.4 (0.6) | 0.454 |
| **EMDA** | **Overall** | **T1**  **Low adherence** | **T2**  **Medium adherence** | **T3**  **High adherence** | *p value* |
| Participants, n | 79 | 26 | 28 | 25 |  |
| *Servings/day* | | | | | |
| Fruits & vegetables | 3.3 (2.7) | 2.2 (1.8) | 2.7 (2.1) | 5.0 (3.2) | <0.001 |
| Pulses | 0.4 (0.6) | 0.1 (0.2) | 0.4 (0.6) | 0.7 (0.7) | 0.007 |
| Wholegrains | 1.0 (1.0) | 0.6 (0.5) | 1.1 (1.1) | 1.4 (1.0) | 0.016 |
| FPF (savoury) | 0.7 (0.8) | 1.0 (0.9) | 0.8 (0.9) | 0.4 (0.5) | 0.006 |
| FPF (sweet) | 1.2 (1.0) | 1.7 (1.1) | 1.0 (0.9) | 0.7 (0.9) | 0.001 |
| Red meat | 0.3 (0.5) | 0.4 (0.5) | 0.4 (0.6) | 0.1 (0.2) | 0.005 |
| Processed meat | 0.7 (1.0) | 0.7 (0.6) | 1.0 (1.5) | 0.3 (0.5) | 0.016 |
| Sugar-sweetened drinks | 0.4 (0.6) | 0.7 (0.8) | 0.3 (0.6) | 0.2 (0.5) | 0.001 |
| Alcoholic drinks | 0.4 (0.6) | 0.4 (0.7) | 0.2 (0.3) | 0.5 (0.8) | 0.315 |

Values are presented as means (SD), servings/day. See **Supplementary Table 3** for details on food group classifications. An X2 test, one-way ANOVA or Kruskal-Wallis test was carried out as appropriate (significance p < 0.05). EMDA: Ecological Momentary Dietary Assessment; FFQ: Food frequency questionnaire; FPF: Fast and processed foods; SD: Standard deviation; T: Tertile.

**Supplementary Table 6.** Distribution and agreement between scores of each WCRF/AICR recommendation via Screener and *Validated Score-FFQ* (n=101)

|  | **WCRF/AICR Screener** | | | ***Validated Score-FFQ*** | | | **Cross - classification** | **CWK** |
| --- | --- | --- | --- | --- | --- | --- | --- | --- |
| **Score** | **0** | **0.5** | **1** | **0** | **0.5** | **1** |  |  |
|  | n (%) | n (%) | n (%) | n (%) | n (%) | n (%) | (%) | (κ) |
| Body composition | 4 (4.0) | 19 (18.8) | 78 (77.2) | 2 (2.0) | 13 (12.9) | 86 (85.2) | 94.1 | 0.67 |
| Physical activity | 20 (19.8) | 11 (10.9) | 70 (69.3) | 8 (7.9) | 58 (57.4) | 35 (34.7) | 65.8 | 0.15 |
| Plant-based foods# | 22 (21.8) | 64 (63.4) | 15 (14.9) | 12 (11.9) | 49 (48.5) | 40 (39.6) | 80.7 | 0.46 |
| Fruit & vegetables | 24 (23.8) | 58 (57.4) | 19 (18.8) | 8 (7.9) | 32 (31.7) | 61 (60.4) | 70.3 | 0.29 |
| Pulses | 13 (12.9) | 59 (58.4) | 29 (28.7) | 23 (22.8) | 31 (30.7) | 47 (46.5) | 79.2 | 0.47 |
| Wholegrains | 56 (55.5) | 24 (23.8) | 21 (20.8) | 32 (31.7) | 40 (39.6) | 29 (29.7) | 71.3 | 0.36 |
| Fast and processed foods | 33 (32.7) | 29 (28.7) | 39 (38.6) | 85 (84.2) | 14 (13.9) | 2 (2.0) | 55.9 | 0.13 |
| Red and processed meat | 46 (45.5) | 19 (18.8) | 36 (35.6) | 71 (70.3) | 8 (7.9) | 22 (21.8) | 77.7 | 0.52 |
| Sugar-sweetened drinks | 4 (4.0) | 48 (47.5) | 49 (48.5) | 6 (5.9) | 83 (82.2) | 12 (11.9) | 75.7 | 0.17 |
| Alcoholic drinks | 1 (1.0) | 65 (64.4) | 35 (34.7) | 1 (1.0) | 77 (76.2) | 23 (22.8) | 90.1 | 0.54 |

*Validated Score-FFQ* refers to the WCRF/AICR Score constructed using valid data on body composition, physical activity and diet using measured anthropometry (stadiometer and measuring tape), accelerometry (accelerometer) and food intake (FFQ). #Scores for plant-based foods (computed as the mean of the individual scores assigned to “Fruit & vegetables”, “Pulses” and “Wholegrains”) were rounded to 0.5 in this table. Cross-classification (%) was calculated as the percentage of participants scoring the same using the WCRF/AICR Screener and validated methods. CWK statistic (κ values) indicates: >0.8 almost perfect agreement, 0.61–0.80 substantial agreement, 0.41–0.60 moderate agreement, 0.21–0.40 fair agreement, and ≤0.20 slight agreement. CWK: Cohen’s weighted kappa; FFQ: Food frequency questionnaire; WCRF/AICR: World Cancer Research Fund/American Institute for Cancer Research.

**Supplementary Table 7.** Matrix with all of the networks’ partial connections between variables

|  | **Fruit and vegetables** | **Legumes** | **Wholegrains** | **Fast and processed food-savoury** | **Fast and processed food-sweet** | **Red meat** | **Processed meat** | **Sugar-sweetened drinks** | **Alcohol** |
| --- | --- | --- | --- | --- | --- | --- | --- | --- | --- |
| **Fruit and vegetables** | - |  |  |  |  |  |  |  |  |
| **Legumes** | 0.22 | - |  |  |  |  |  |  |  |
| **Wholegrains** | 0.21 | 0.00 | - |  |  |  |  |  |  |
| **Fast and processed food-savoury** | -0.04 | 0.00 | 0.00 | - |  |  |  |  |  |
| **Fast and processed food-sweet** | 0.00 | 0.00 | 0.00 | 0.26 | - |  |  |  |  |
| **Red meat** | 0.00 | -0.23 | 0.00 | 0.00 | 0.00 | - |  |  |  |
| **Processed meat** | -0.02 | 0.00 | 0.00 | 0.16 | 0.03 | 0.19 | - |  |  |
| **Sugar-sweetened drinks** | -0.10 | -0.06 | -0.24 | 0.15 | 0.15 | 0.00 | 0.14 | - |  |
| **Alcohol** | 0.00 | 0.00 | 0.00 | 0.00 | 0.00 | -0.04 | 0.00 | 0.00 | - |

The matrix displays the partial correlation coefficients between all pairs of variables included in the diet dimension of the WCRF/AICR Screener. Each value represents the unique association between two variables, after adjusting for all other variables in the network. These estimates correspond to the edge weights in the diet-related network model and were obtained using the Extended Bayesian Information Criterion graphical LASSO (EBICglasso), which applies regularization to enhance model sparsity and stability.

**Supplementary Table 8.** Network edge weights with bootstrapped confidence intervals

| **Node 1** | **Node 2** | **Edge weight** | **Bootstrapped mean** | **Bootstrapped SD** | **Bootstrapped 95% CI** |
| --- | --- | --- | --- | --- | --- |
| **Wholegrains** | **Sugar-sweetened drinks** | -0.30 | -0.32 | 0.10 | -0.54, -0.13 |
| **Fast and processed food-savoury** | **Fast and processed food-sweet** | 0.30 | 0.30 | 0.09 | 0.11, 0.47 |
| **Legumes** | **Red meat** | -0.27 | -0.28 | 0.11 | -0.48, -0.04 |
| **Red meat** | **Processed meat** | 0.24 | 0.25 | 0.09 | 0.06, 0.41 |
| **Fruit and vegetables** | **Legumes** | 0.24 | 0.23 | 0.10 | 0.03, 0.40 |
| **Fruit and vegetables** | **Wholegrains** | 0.20 | 0.20 | 0.08 | 0.03, 0.36 |
| **Fast and processed food-savoury** | **Sugar-sweetened drinks** | 0.18 | 0.18 | 0.10 | -0.00, 0.37 |
| **Fast and processed food-savoury** | **Processed meat** | 0.18 | 0.18 | 0.09 | 0.00, 0.34 |
| **Processed meat** | **Sugar-sweetened drinks** | 0.18 | 0.18 | 0.09 | 0.00, 0.36 |
| **Fruit and vegetables** | **Sugar-sweetened drinks** | -0.16 | -0.14 | 0.09 | -0.32, 0.00 |
| **Fast and processed food-sweet** | **Sugar-sweetened drinks** | 0.15 | 0.16 | 0.09 | 0.00, 0.35 |
| **Red meat** | **Alcohol** | -0.08 | -0.11 | 0.09 | -0.30, -0.00 |
| **Sugar-sweetened drinks** | **Alcohol** | 0.06 | 0.07 | 0.07 | 0.00, 0.24 |
| **Fruit and vegetables** | **Fast and processed food-savoury** | -0.06 | -0.07 | 0.07 | -0.23, 0.00 |
| **Legumes** | **Sugar-sweetened drinks** | -0.05 | -0.05 | 0.07 | -0.20, 0.00 |
| **Legumes** | **Wholegrains** | 0.00 | 0.03 | 0.05 | 0.00, 0.18 |
| **Legumes** | **Fast and processed food-savoury** | 0.00 | -0.01 | 0.04 | -0.11, 0.00 |
| **Wholegrains** | **Fast and processed food-savoury** | 0.00 | 0.02 | 0.05 | 0.00, 0.18 |
| **Fruit and vegetables** | **Fast and processed food-sweet** | 0.00 | 0.00 | 0.04 | -0.10, 0.12 |
| **Legumes** | **Fast and processed food-sweet** | 0.00 | -0.01 | 0.03 | -0.11, 0.00 |
| **Wholegrains** | **Fast and processed food-sweet** | 0.00 | 0.03 | 0.07 | 0.00, 0.23 |
| **Fruit and vegetables** | **Red meat** | 0.00 | 0.00 | 0.04 | -0.08, 0.12 |
| **Wholegrains** | **Red meat** | 0.00 | 0.00 | 0.04 | -0.10, 0.11 |
| **Fast and processed food-savoury** | **Red meat** | 0.00 | -0.02 | 0.05 | -0.16, 0.00 |
| **Fast and processed food-sweet** | **Red meat** | 0.00 | 0.03 | 0.06 | 0.00, 0.20 |
| **Fruit and vegetables** | **Processed meat** | 0.00 | -0.03 | 0.05 | -0.19, 0.00 |
| **Legumes** | **Processed meat** | 0.00 | -0.02 | 0.05 | -0.17, 0.03 |
| **Wholegrains** | **Processed meat** | 0.00 | 0.04 | 0.08 | 0.00, 0.27 |
| **Fast and processed food-sweet** | **Processed meat** | 0.00 | 0.02 | 0.06 | 0.00, 0.17 |
| **Red meat** | **Sugar-sweetened drinks** | 0.00 | 0.00 | 0.03 | -0.07, 0.07 |
| **Fruit and vegetables** | **Alcohol** | 0.00 | 0.00 | 0.03 | -0.07, 0.09 |
| **Legumes** | **Alcohol** | 0.00 | 0.01 | 0.04 | -0.02, 0.12 |
| **Wholegrains** | **Alcohol** | 0.00 | -0.03 | 0.06 | -0.18, 0.00 |
| **Fast and processed food-savoury** | **Alcohol** | 0.00 | 0.04 | 0.06 | 0.00, 0.20 |
| **Fast and processed food-sweet** | **Alcohol** | 0.00 | -0.03 | 0.07 | -0.23, -0.00 |
| **Processed meat** | **Alcohol** | 0.00 | 0.01 | 0.04 | -0.07, 0.15 |

Edge weights represent regularized partial correlation coefficients estimated using the Extended Bayesian Information Criterion graphical LASSO (EBICglasso). Bootstrapped means, standard deviations, and 95% confidence intervals were obtained using nonparametric bootstrap analyses. Positive values indicate positive conditional associations, whereas negative values indicate negative conditional associations, after adjusting for all other variables in the network.

**Supplementary Table 9.** Centrality indices and stability coefficients of the dietary network

| **Node** | **Strength** | **Closeness** | **Betweenness** |
| --- | --- | --- | --- |
| **Sugar-sweetened drinks** | 1.081 | 0.016 | 14 |
| **Fast and processed food-savoury** | 0.724 | 0.013 | 6 |
| **Fruit and vegetables** | 0.651 | 0.013 | 6 |
| **Processed meat** | 0.603 | 0.015 | 12 |
| **Red meat** | 0.602 | 0.014 | 16 |
| **Legumes** | 0.562 | 0.012 | 8 |
| **Wholegrains** | 0.497 | 0.013 | 0 |
| **Fast and processed food-sweet** | 0.448 | 0.011 | 0 |
| **Alcohol** | 0.145 | 0.007 | 0 |

Strength, closeness, and betweenness were estimated from the EBICglasso network. Centrality stability coefficients obtained using case-dropping bootstrap analyses were 0.205 for strength, 0.000 for closeness, and 0.000 for betweenness. Given the low stability coefficients, particularly for closeness and betweenness, centrality indices should be interpreted with caution.

**Supplementary Figure 1.** Study scheme


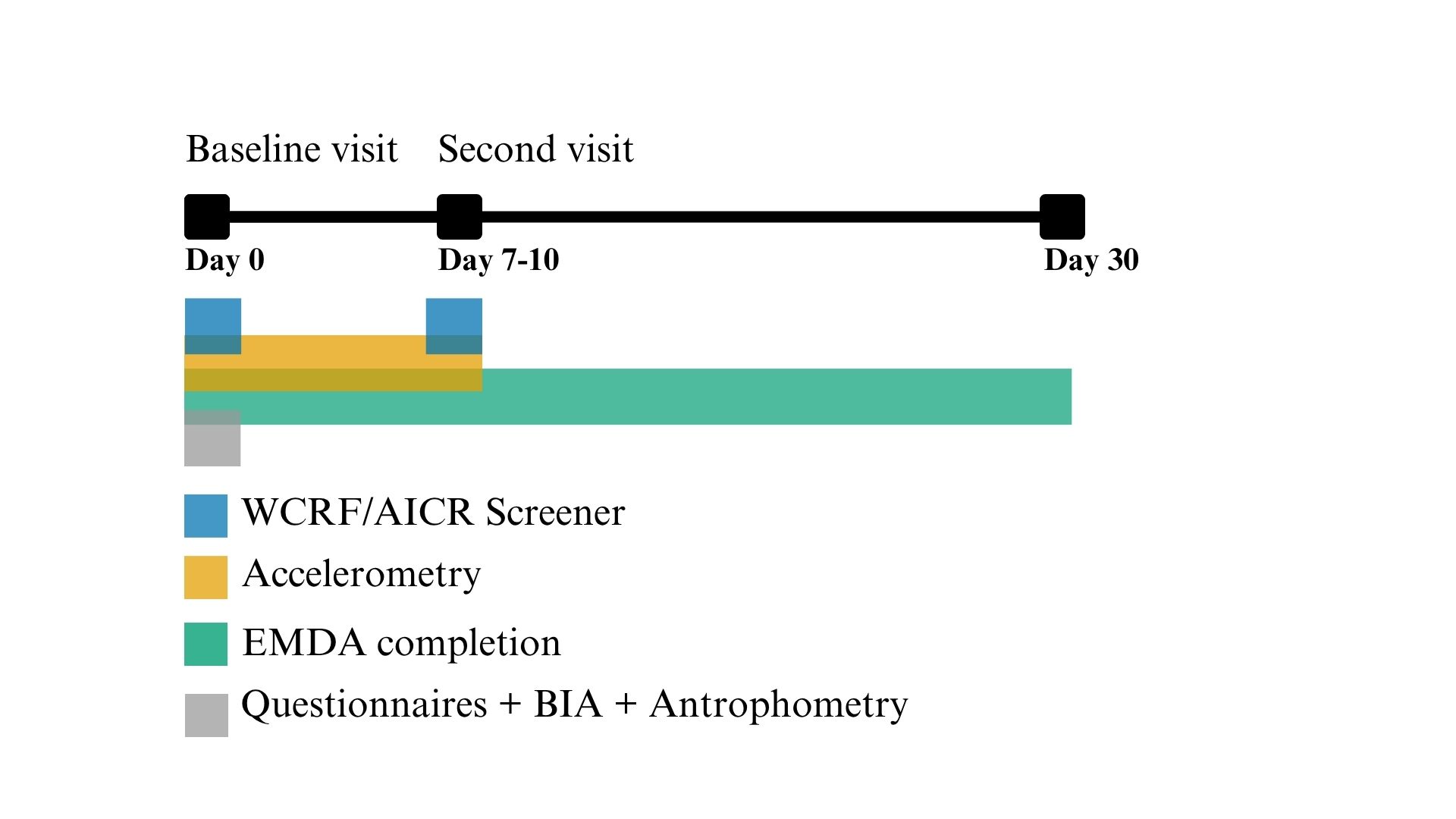


BIA: Bioelectrical impedance analysis; EMDA: Ecological Momentary Dietary Assessment; WCRF/AICR: World Cancer Research Fund/American Institute for Cancer Research.

**Supplementary Figure 2.** Flowchart of the study


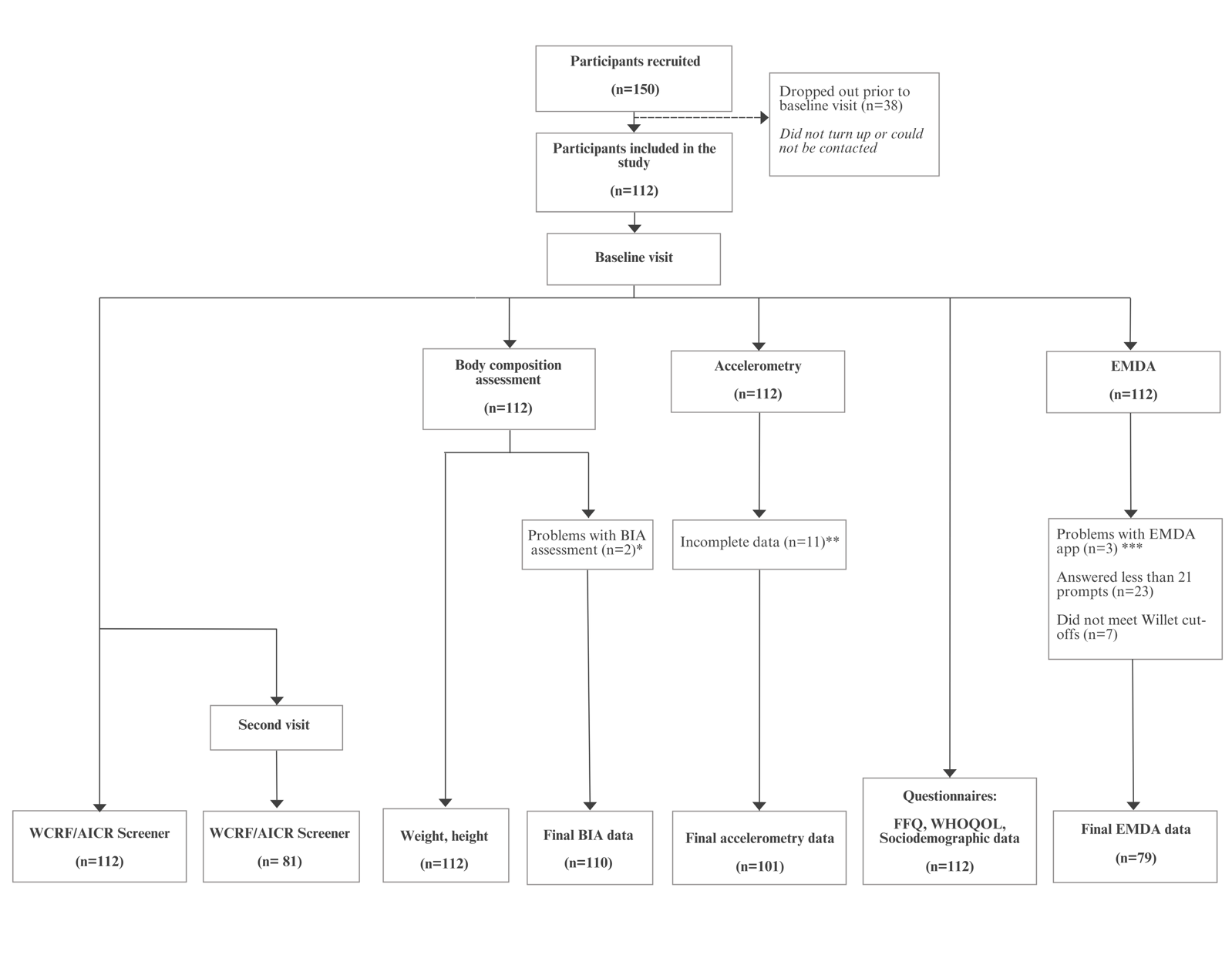


***** BIA was not feasible for two participants; ** Eleven participants were excluded from the accelerometer analysis due to incomplete data, resulting from either reported skin irritation or device malfunction; *** Problems with EMDA app included: lack of space on the participant’s mobile phone to install the EMDA app or could not set up notifications. Did not meet the Willet cut-off criteria included 1 under-reporter and 6 over-reporters. BIA: Bioelectrical impedance analysis; EMDA: Ecological Momentary Dietary Assessment; FFQ: Food frequency questionnaire; WCRF/AICR: World Cancer Research Fund/American Institute for Cancer Research; WHOQOL: WHOQOL-BREF (World Health Organization quality of life questionnaire).

**Supplementary Figure 3.** Bland-Altman Plot for the agreement of the scores derived from the WCRF/AICR Screener and *Validated Score-EMDA*


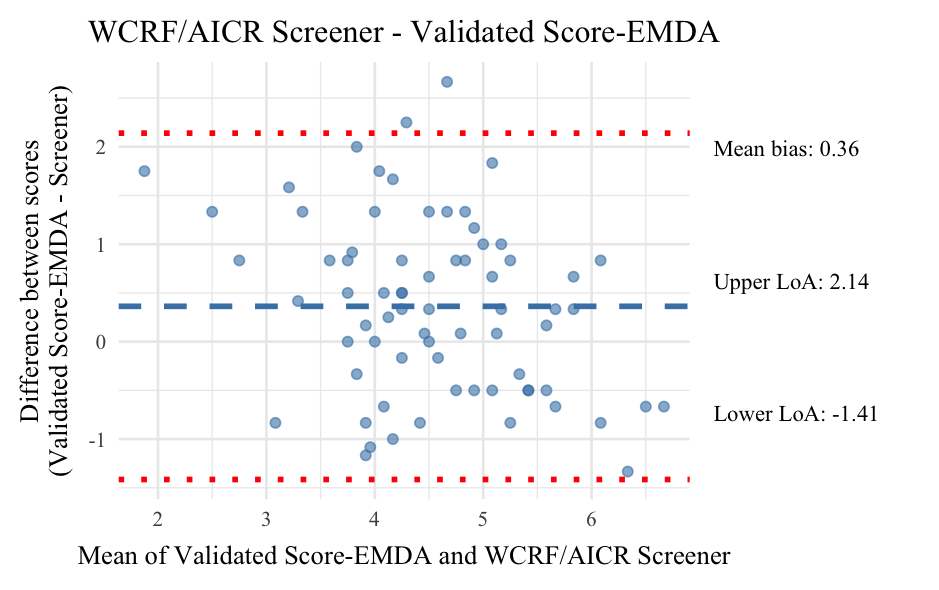


Total number of participants which completed *Validated Score-EMDA was 71.* *Validated Score-EMDA*: Refers to the WCRF/AICR Score constructed using valid data on body composition, physical activity and diet using directly measured anthropometry, accelerometer and EMDA. The Bland–Altman difference was calculated as *Validated Score-EMDA* minus WCRF/AICR Screener score. Mean difference = 0.36 (SD 0.91; 95% CI 0.15 to 0.58); limits of agreement = -1.41 to 2.14. The 95% CIs for the lower and upper limits of agreement were -1.78 to -1.05 and 1.77 to 2.51, respectively. Proportional bias regression: intercept = 1.8 (SE 0.52; 95% CI 0.76 to 2.83; p = <0.001), slope = -0.32 (SE 0.11; 95% CI -0.54 to -0.09; p = 0.006), R² = 0.1. EMDA: Ecological Momentary Dietary Assessment; WCRF/AICR: World Cancer Research Fund/American Institute for Cancer Research.

**Supplementary Figure 4.** Bland-Altman Plot for the agreement of the scores derived from the WCRF/AICR Screener and *Validated Score-FFQ*


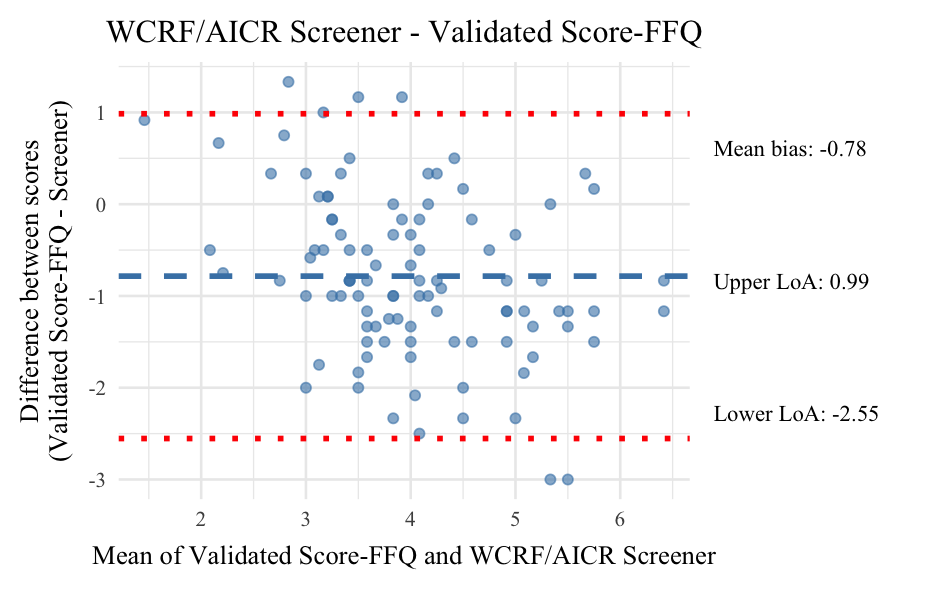
Total number of participants which completed *Validated Score-FFQ* was 101. *Validated Score-FFQ*: Refers to the WCRF/AICR Score constructed using valid data on body composition, physical activity and diet using directly measured anthropometry, accelerometer and FFQ. The Bland–Altman difference was calculated as Validated Score-FFQ minus WCRF/AICR Screener score. Mean difference = -0.78; limits of agreement = -2.55 to 0.99. Proportional bias regression: intercept = 0.56 (SE 0.37; 95% CI -0.18 to 1.29; p = 0.134), slope = -0.33 (SE 0.09; 95% CI -0.51 to -0.16; p = <0.001), R² = 0.12. FFQ: Food frequency questionnaire; WCRF/AICR: World Cancer Research Fund/American Institute for Cancer Research.

**Supplementary Figure 5.** Edge weight stability analysis in the network: comparison of sample estimates and bootstrap results


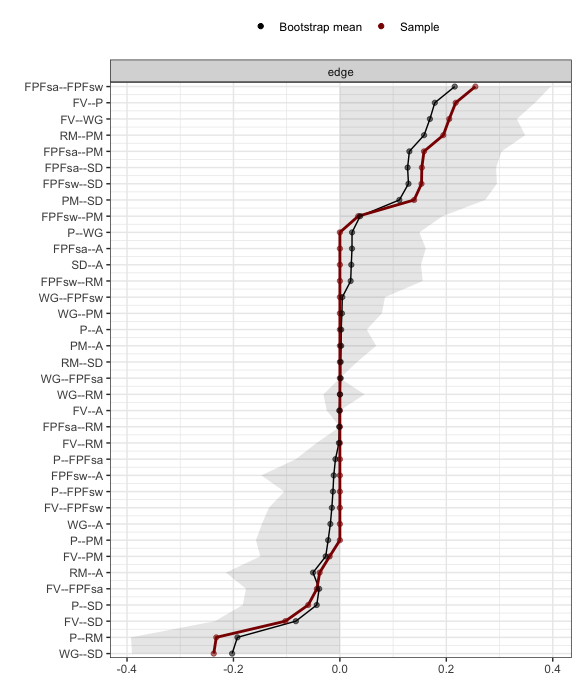


The grey area represents the confidence interval around the bootstrapped mean. In general, the proximity of the “Sample” edge weight to the “Bootstrap mean” provides an indication of edge stability: smaller distances between these points suggest that the edge weight is less sensitive to variations in the sample data. A: Alcoholic drinks; FPFsa: Fast and processed food – savoury; FPFsw: Fast and processed food – sweet; FV: Fruit and vegetables, P: Pulses; PM: Processed meat; RM: Red meat; SD: Sugar-sweetened drinks; WG: Wholegrains.
